# Schisandrin A regulates the Nrf2 signaling pathway and inhibits NLRP3 inflammasome activation to interfere with pyroptosis in a mouse model of COPD

**DOI:** 10.1186/s40001-023-01190-8

**Published:** 2023-07-03

**Authors:** Jiamin Zeng, Sida Liao, Zhu Liang, Caiping Li, Yuewen Luo, Kexin Wang, Dapeng Zhang, Lan Lan, Suzhen Hu, Wanyan Li, Ran Lin, Zichen Jie, Yuanlong Hu, Shiting Dai, Zhimin Zhang

**Affiliations:** 1 The First Affiliated Hospital of Guangzhou Medical University, Guangzhou, China; 2 Guangzhou Medical University, Guangzhou, China; 3 Huangdao District Chinese Medicine Hospital, Qingdao, China; 4 The Second Clinical College, Guangzhou University of Chinese Medicine, Guangzhou, China

## Abstract

Chronic obstructive pulmonary disease (COPD) is a serious chronic lung disease. Schisandrin A (SchA) is one of the most important active ingredients in *Schisandra chinensis* and has been used to treat various lung diseases in several countries. Here, we studied the pharmacological effect of SchA on airway inflammation induced by cigarette smoke (CS) and explored the therapeutic mechanism of SchA in COPD model mice. Our results showed that SchA treatment significantly improved the lung function of CS-induced COPD model mice and reduced the recruitment of leukocytes and hypersecretion of interleukin-6 (IL-6), interleukin-1β (IL-1β) and tumor necrosis factor α (TNF-α) in bronchoalveolar lavage fluid (BALF). H&E staining showed that SchA treatment could effectively reduce emphysema, immune cell infiltration and airway wall destruction. In addition, we found that SchA treatment can stimulate the expression of heme oxygenase-1 (HO-1) through the nuclear factor-erythroid 2-related factor (Nrf2) pathway, significantly reduce oxidative stress, increase catalase (CAT) and superoxide dismutase (SOD) levels, and suppress the level of malondialdehyde (MDA) in COPD model mice. Moreover, SchA treatment suppressed the generation of the NLRP3/ASC/Caspase1 inflammasome complex to inhibit the inflammatory response caused by IL-1β and IL-18 and pyroptosis caused by GSDMD. In conclusion, our study shows that SchA treatment can inhibit the production of ROS and the activation of the NLRP3 inflammasome by upregulating Nrf-2, thereby producing anti-inflammatory effects and reducing lung injury in COPD model mice. More importantly, SchA exhibited similar anti-inflammatory effects to dexamethasone in COPD model mice, and we did not observe substantial side effects of SchA treatment. The high safety of SchA makes it a potential candidate drug for the treatment of COPD.

## Introduction

Chronic obstructive pulmonary disease (COPD) is a common, preventable and treatable disease characterized by persistent respiratory symptoms and airflow limitation due to airway and alveolar abnormalities. COPD is usually associated with significant exposure to toxic particles and gases and is currently the third leading cause of death in China [1]. The prevalence of COPD in people over 40 years old is 10% worldwide [2] and more than 13% in China, representing a total of 99.9 million people in the country, which causes a significant social burden.

Nuclear factor erythroid 2-related factor 2 (Nrf2) is an important endogenous antioxidant transcription factor in the body. When activated, Nrf2 translocates into the nucleus and binds to antioxidant response elements (AREs) in the promoter region of target genes; its deletion or activation can cause intracellular oxidative-antioxidative imbalance, and the persistent presence of reactive oxygen species (ROS) leads to reduced cellular repair. In severe cases, cells undergo irreversible senescence or even cell death. Activation of the Nrf2 pathway can reduce the degree of oxidative stress in COPD, ameliorate lung tissue damage, and slow the progression of COPD. Numerous studies have shown that regulating the Nrf2 pathway plays a very important role in preventing pyroptosis [3–6].

Pyroptosis is different from other forms of programmed cell death, which mainly depend on caspase-1, 4, 5 and 11. The basic characteristics of pyroptosis are cell swelling, formation of plasma membrane pores (dissolution) and release of the proinflammatory cytokine interleukin, but it is still a new cell death mode that causes cell death represented by the NLR family [7]. In the NLR family, the NLRP3 inflammasome is widely studied and well known by researchers because of its close relationship with clinical practice. NLRP3 inflammasome activation plays an important role in the formation of early inflammation, and it is regulated by damage-associated molecular patterns in vivo. NLRP3 inflammatory corpuscles are composed of three parts: receptor (NLRP3), apoptosis-related speckle-like protein (ASC) and effector protein (activated caspase-1). Activation in the classical pyroptosis pathway depends on caspase-1 cutting the key mediator Gasdermin D (GSDMD), and the amino terminal domain of GSDMD can cause a series of inflammatory cascade reactions by activating the NLRP3 inflammasome in the pyroptosis pathway [8, 9]. Moreover, it can activate a variety of immune cells, including macrophages, neutrophils and dendritic cells, to aggregate and release more inflammatory factors, thereby regulating innate and adaptive immunity and mediating pyroptosis. Regulation of the Nrf2 pathway can reduce the level of intracellular ROS, which may inhibit the activation of the NLRP3 inflammasome.

Schisandrin A (SchA) is a diphenyl cyclooctadiene lignan isolated from the fruit of *Schisandra chinensis* that has various pharmacological effects [10]. SchA is widely used in traditional Chinese medicine as a cough suppressant, tonic and sedative and is also a component of dietary supplement products in the United States [11, 12]. Some scholars have shown that SchA has an anti-inflammatory effect in acute respiratory distress syndrome [13]. Therefore, we hypothesized that SchA might also regulate inflammation in COPD. The regulation of the Nrf2 pathway by dexamethasone (Dex) has been confirmed by other scholars [14], but whether the underlying mechanism of its effective treatment of COPD is to interfere with pyroptosis by inhibiting NLRP3 remains to be explored.

The main purpose of this study was to clarify the mechanism by which SchA interferes with pyroptosis in the lungs of a COPD mouse model. Therefore, we proposed a scientific hypothesis: SchA plays a role in the treatment of COPD by regulating the Nrf2 pathway, accelerating the clearance of ROS in the lung, and inhibiting the activation of the NLRP3 inflammasome, thereby interfering with cell pyroptosis.

## Materials and methods

### Construction of the mouse COPD model and treatments

All experimental protocols were approved by the Ethics Committee of Guangzhou Medical University. Cigarette smoke (CS) is a major factor driving the onset and progression of COPD [15]. Studies have shown that CS can accelerate lung inflammation in both mice and humans [16]. Therefore, we followed the method reported in a paper on animal model construction. Briefly, healthy C57BL/6J male mice (6–8 weeks old, wild-type) were purchased from Vital River Animals and housed in a specific pathogen-free facility with free access to food and water. Mice were placed in a chamber and exposed to CS from week 0 to week 30 (10 cigarettes/1, 2 h/session, twice/day, 6 days/week) after a 2-week acclimation period. The weight of each mouse was measured every Saturday from 8 to 9 am using the same scale, and weight gain was calculated from the weekly weight data. The cigarettes used in this study were commercial Meihua brand cigarettes (Tobacco Industry, China). Each cigarette produces 11 mg of tar, 0.9 mg of nicotine and 12 mg of carbon monoxide. Thirty mice were randomly assigned to the following groups (total of 30 mice, 6 per group) and treated with drugs from 24 to 30 weeks. The doses of the drugs used were based on conventional doses used by other scholars [14, 17]. Schisandrin A was purchased from MedChemExpress.

**Fig. 1:**
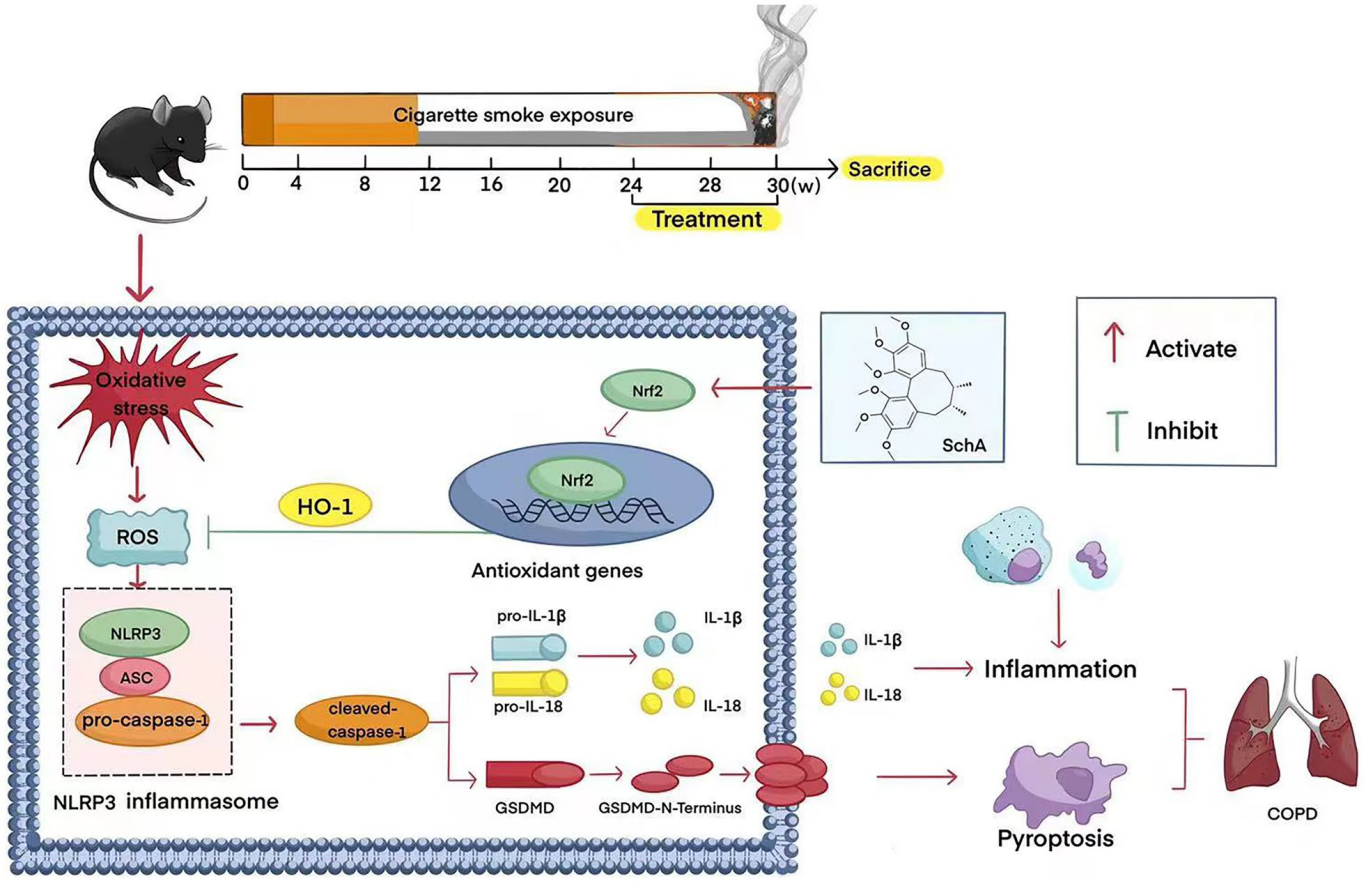
Schematic diagram of *SchisandrinA* regulating Nrf2 to inhibit NLRP3 interference with cell pyroptosis

1. Control group: Mice were only exposed to room air and had free access to sterile water and food. In this group, 10 mL/kg physiological saline was given by gavage.
2. CS group: Mice were subjected to CS as previously described and had free access to water and food. In this group, 10 mL/kg physiological saline was given by gavage. The whole course lasts for 30 weeks.
3. CS + Dex group: The smoke exposure method was the same as that of the CS group. At the 24th week of smoke exposure, mice received Dex (0.6 mg/kg/d treatment) by gavage for 6 weeks according to other scholars [14], along with smoke exposure. The whole course lasts for 30 weeks.
4. CS + SchA group: The smoke exposure method was the same as that of the CS group. At the 24th week of smoke exposure, mice received Sch A by gavage for 6 weeks, along with smoke exposure. The whole course lasts for 30 weeks.
5. CS + SchA + Dex group: The smoke exposure method was the same as that of the CS group. The administration plan of Dex and SchA was as described in the third and fourth groups. The two drugs were administered at the same time in weeks 24–30 (see Fig. 1 for details)

In addition to modeling, as shown in Fig. 1, we conducted pharmacological exploration of the therapeutic mechanism of SchA. In summary, our study discussed the efficacy of SchA in the treatment of COPD model mice in vivo and compared it with the efficacy of Dex treatment. We further discussed its therapeutic pharmacological mechanism. We propose that SchA administration can treat COPD by regulating the Nrf2 signaling pathway and inhibiting NLRP3 activation, as shown in Fig. 1.

### Lung function tests

Mice in each group were anesthetized with 2% avertin (17.5 μL/g intraperitoneally) and then tracheotomized and intubated. Mechanical ventilation was performed using the Pulmonary Maneuver System (Buxco Research Systems, Wilmington, NC, USA) according to the manufacturer's instructions. After assessing lung function metrics, we collected two lung tissue samples for further analysis.

### Bronchoalveolar lavage fluid (BALF) analysis

BALF was collected from each group of mice. The right lung was slowly injected with 0.3 mL of physiological saline 3 times, the recovery rate was over 80%, and BALF was collected in test tubes and placed on ice. After BALF centrifugation (300 × g, 10 min, 4 °C), the supernatant was stored at − 80 °C for subsequent cytokine assays. Then, the cell pellet was resuspended in 1 mL phosphate buffer saline (PBS), 10 μL was collected, and the total number of cells was counted using a hemocytometer. The remaining BALF was centrifuged again (300 × g, 5 min, 4 °C) to pick up the bottommost cells and spread them evenly on the microscope slide. Then, the cells were fixed and subjected to Diff Quik (BASO Biology, China) staining for identification and quantification of neutrophils and macrophages. The proportion of inflammatory cells was quantitatively analyzed. In addition, ELISA kits (Invitrogen, China) were used to analyze the BALF supernatant, and the levels of the inflammatory factors TNF-α, IL-1β and IL-6 were measured.

### Histopathological examination and morphological analysis

The left lung tissue samples from mice were first fixed in 4% paraformaldehyde for 24 h. The fixed lung tissue was then embedded in paraffin, cut into 4 µm thick sections, and stained with hematoxylin and eosin (Servicebio, China). H&E staining images were analyzed and measured using ImageJ software. We used a pathological scoring system as a semiquantitative analysis to assess the severity of CS-induced pulmonary inflammation in mice, defined as follows: 0 = no detectable inflammation; 1 = mild inflammation: occasional accumulation of inflammatory cells in bronchial or vessel walls and alveolar septa; 2 = moderate inflammation: most alveoli, bronchi, or blood vessel walls have 1 to 5 layers of inflammatory cells; and 3 = severe inflammation: most walls of bronchi or blood vessels and alveoli are separated by five or more layers of inflammatory cells [18].

Next, emphysema lesions were quantified by the mean linear intercept (MLI), which is the total length of the intersection line (L) in each random field divided by the number of alveoli (NA) in each field that crosses the intersection line. The MLI was calculated by the following formula: MLI = L/NA.

To calculate the tracheal wall thickness, a complete small trachea cross-section was selected for measuring the total bronchial wall area (WAt, μm^2^) and the perimeter of the bronchial basement membrane (Pbm, μm). The WAt/Pbm ratio was used to evaluate airway remodeling [19].

For immunohistochemical evaluation of the tissue, lung tissue sections were dewaxed in water, washed after microwave repair, and sealed with goat serum. The sections were then stained with primary antibodies against NRF2 (Servicebio, China) and NLRP3 (R&D Systems, America) at 4 °C overnight. After 1 h of incubation with secondary antibodies, nuclei were counterstained with DAPI for 5 min. Then, the samples were observed with a microscope.

### Western blotting and quantitative analysis

To determine the expression levels of Nrf2, NLRP3 and their downstream related proteins in lung tissue, we conducted a Western blotting (WB) experiment. The right lung tissue was homogenized with phenylmethylsulfonyl fluoride (PMSF) in ice-cold RIPA lysis buffer (Beyotime Biotechnology, China), and the supernatant was obtained by centrifugation (13,000 rpm, 30 min, 4 °C). Then, the total protein concentration in the supernatant was measured using a bicinchoninic acid protein (BCA) assay kit (Vazyme, China). Equal amounts of proteins were separated by SDS–polyacrylamide gel electrophoresis and transferred to PVDF membranes. The membranes were blocked in blocking solution (5% dry skim milk) for 24 h at 4 °C. Then, the PVDF membranes were incubated with specific primary antibodies for 24 h at 4 °C. Next, the secondary antibody was added and incubated for 2 h at room temperature. Finally, specific proteins were visualized with an ECL reagent in the Tanon5200 imaging system and semiquantitatively analyzed based on the grayscale values of the bands. Protein from one animal per group was used for a single WB experiment. Primary antibodies against NLRP3 and ASC were obtained from Cell Signaling, Caspase1 was obtained from ABclonal, and Nrf2, HO-1, IL-1β, IL18, GSDMD and β-actin were obtained from Abcam and used at a ratio of 1:1000.

### Measuring oxidative stress-related proteins and molecules

We used a fluorescent dye probe to measure ROS production in cells. Briefly, fresh right lung tissue was embedded in OCT compound, sectioned, and stained with dihydroethidium (DHE) to measure ROS levels. After DHE enters the cell, it is oxidized by superoxide and emits red fluorescence, which is detected by a fluorescence microscope (Leica, Germany). Based on 6 mice in each group, three slices were randomly taken, with a total of 18 pictures in each group, and then the area where the relative fluorescence intensity value was selected was measured and analyzed with ImageJ software (v1.8.0). For the detection of malondialdehyde (MDA), catalase (CAT) and superoxide dismutase (SOD) levels, we collected the right lung of each group of mice and used detection kits following the manufacturer’s protocol (Beyotime, China).

### Statistical analysis

All data are from three or more independent experiments and were analyzed by SPSS 25.0 statistical software (IBM, Armonk, NY, USA). The results of the normality test are expressed as the mean ± standard error (SEM), and the failed data are expressed as the median ± quartile (IQR). Normally distributed data among the groups were analyzed for statistical significance using one-way analysis of variance (ANOVA) and Tukey's post hoc test for multiple comparisons if the population variance was equal; otherwise, Games–Howell's post hoc test was used. For nonnormally distributed data, the nonparametric Kruskal‒Wallis test was used for group comparisons among the different groups. A value of *p* < 0.05 was considered statistically significant.

## Results

### SchA treatment improves lung function in CS-induced COPD model mice

**Fig. 2:**
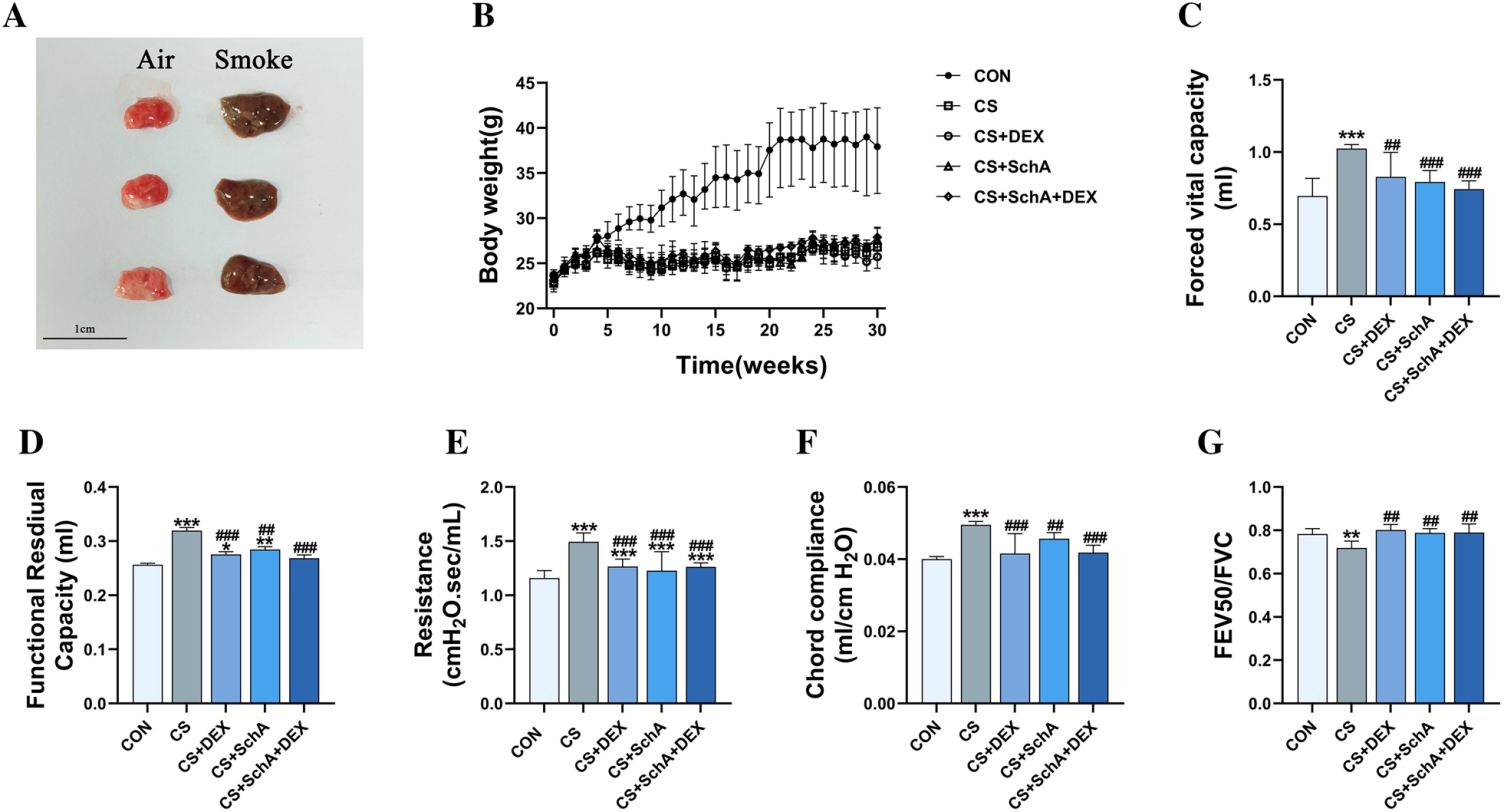
Comparison of lung tissue morphology, body weight and lung function of mice in each group. **A** The lung tissue of the control group mice is on the left, and the lung of the CS-induced COPD group mice is on the right. **B** Mouse weight changes during the experiments. **C** Forced vital capacity. **D** Functional residual capacity. **E** Resistance. **F** Chord compliance. **G** FEV50/FVC. *n* = 6 mice per group. Data are shown as the mean ± SEM (**D**) and median ± IQR (**C**, **E**–**G**). To test for group differences, Games–Howell (**D**) and Kurskal–Wallis (**C**, **E**–**G**). *p < 0.05, **p < 0.01, ***p < 0.001 versus control group, ^#^*p* < 0.05, ^##^*p* < 0.01, ^###^*p* < 0.001 versus CS group

Obviously, the lung tissue that was exposed to CS for a long period displayed significant changes in appearance, including darker tissue color (Fig. 2a). In the process of modeling, we found that mice exposed to CS for a long time showed typical clinical manifestations of COPD. After our long-term observation, we found that compared with the control group, the mice in the CS group were more prone to some specific abnormal symptoms, such as fluffy and yellow hair, irritable personality, and significantly reduced activity. In addition to respiratory manifestations, the typical symptoms of COPD often have systemic manifestations, such as changes in weight. Compared with the control group, the weight of mice exposed to smoke significantly differed (Fig. 2b). Similarly, we found that the body weight of CS mice was significantly lower than that of control mice. However, treatment with DEX and SchA appeared to have little effect on weight.

Notably, there was obvious improvement in the lung function decline caused by CS in the SchA group. Considering the continuous airway inflammation in the lungparenchyma of COPD model mice, which resulted in small airway dysfunction, we carefully evaluated the effect of drugs on the lung function of mice exposed to CS. Compared with the control group, the mice exposed to CS had obvious pulmonary dysfunction, such as airflow restriction. In terms of airway resistance, we observed a significant increase caused by CS. The DEX, SchA, and combination therapy groups all improved this indicator (Fig. 2e). In addition, CS exposure increased chord compliance (Fig. 2f). As shown in Fig. 2c–g, Dex treatment significantly reduced the increase in chord compliance, functional reserve capacity (FRC) and forced vital capacity (FVC) and decreased the trend of deterioration in FEV50/FVC. Interestingly, the addition of SchA does not interfere with the therapeutic effect of Dex. Specifically, compared with Dex treatment alone, the combination treatment of SchA and Dex had a more positive effect on FRC and had no significant difference compared to the control group.

### SchA treatment reduces emphysema and inflammatory cell infiltration in CS-induced COPD model mice

**Fig. 3:**
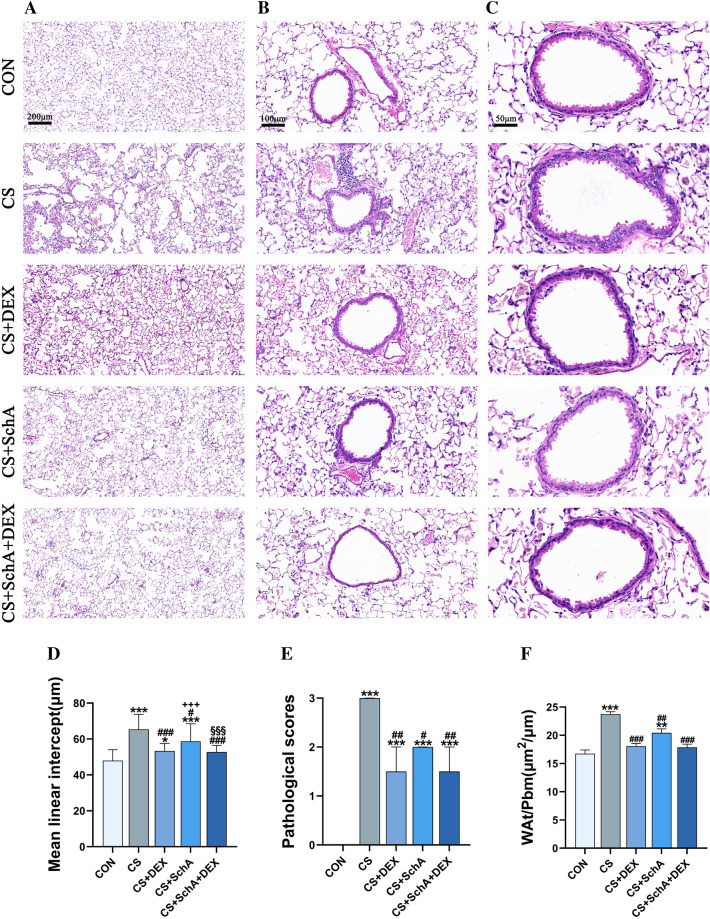
Schisandrin A (SchA) and dexamethasone (Dex) reduce emphysema, inflammatory cell infiltration and small airway remodeling in CS-induced COPD model mice. Lung tissues were stained with H&E. Representative histopathological sections of mouse lungs show **A** expandable alveolar spaces of different degrees (scale bar 200 μm), **B** invasive inflammatory cells (scale bar 100 μm), and **C** thickened bronchial wall (scale bar 50 μm). Different indicators were used to quantify these injuries for statistical analysis, as shown in figure (**D**), the mean linear intercept, which is a quantitative analysis of the severity of emphysema. In addition, quantitative analysis of pulmonary inflammation was determined by **E** pathological score and **F** measurement of the total wall area (Wat/Pbm). *n* = 6 mice per group. Data are shown as the mean ± SEM (**F**) and median ± IQR (**D**–**E**). To test for group differences, Games–Howell (**F**) and Kruskal‒Wallis (**D**–**E**) tests were used. *p < 0.05, **p < 0.01, ***p < 0.001 versus control group, ^#^*p* < 0.05, ^##^*p* < 0.01, ^###^*p* < 0.001 versus CS group, ^+^*p* < 0.05, ^++^*p* < 0.01, ^+++^*p* < 0.001 versus CS + DEX group, ^§^*p* < 0.05, ^§§^*p* < 0.01, ^§§§^*p* < 0.001 versus CS + SchA group

To evaluate the therapeutic effect of drugs on emphysema and inflammatory cell infiltration, we used H&E staining to conduct histopathological examination on the lung tissue of different treatment groups. We found that, compared with the control group, pathological analysis of the lung tissue of CS-induced COPD model mice showed obvious alveolar expansion, alveolar wall damage and fusion, which was consistent with the pathological characteristics of typical emphysema (Fig. 3a).

As shown in Fig. 3a, d, the MLI in the CS group was significantly higher than that in the control group. Furthermore, Dex, SchA, and SchA + Dex can markedly inhibit the lung structure damage caused by CS exposure. Dex treatment was superior in improving MLI over SchA treatment, but SchA treatment still significantly improved MLI compared with that of the CS group, indicating that it also has a therapeutic effect.

In addition, compared with the lung tissue in the control group mice (Fig. 3b, c), the lung tissue in the CS group mice had obvious inflammatory changes that mainly manifested as thickening of the bronchial wall, dysfunction of epithelial cells, and massive infiltration of inflammatory cells. In contrast, the pathological damage was ameliorated in mice treated with SchA or Dex (Fig. 3e, f). Additionally, Dex treatment had an obvious inhibitory effect on emphysema and inflammatory cell infiltration in CS-induced COPD model mice, which further confirmed the clinical anti-inflammatory effect of the drug (Fig. 3e, f). Moreover, there was no significant difference between the use of SchA and Dex individually in increasing airway remodeling and reducing inflammatory cell infiltration, while SchA + Dex showed a similar effect. HE staining results showed typical pathological characteristics of COPD in mice. We found that the lung tissue damage in the SchA group showed an obvious reduction compared to that in the CS group, which significantly differed.

### SchA reduces inflammatory cells and factors in the BALF of CS-induced COPD model mice

**Fig. 4:**
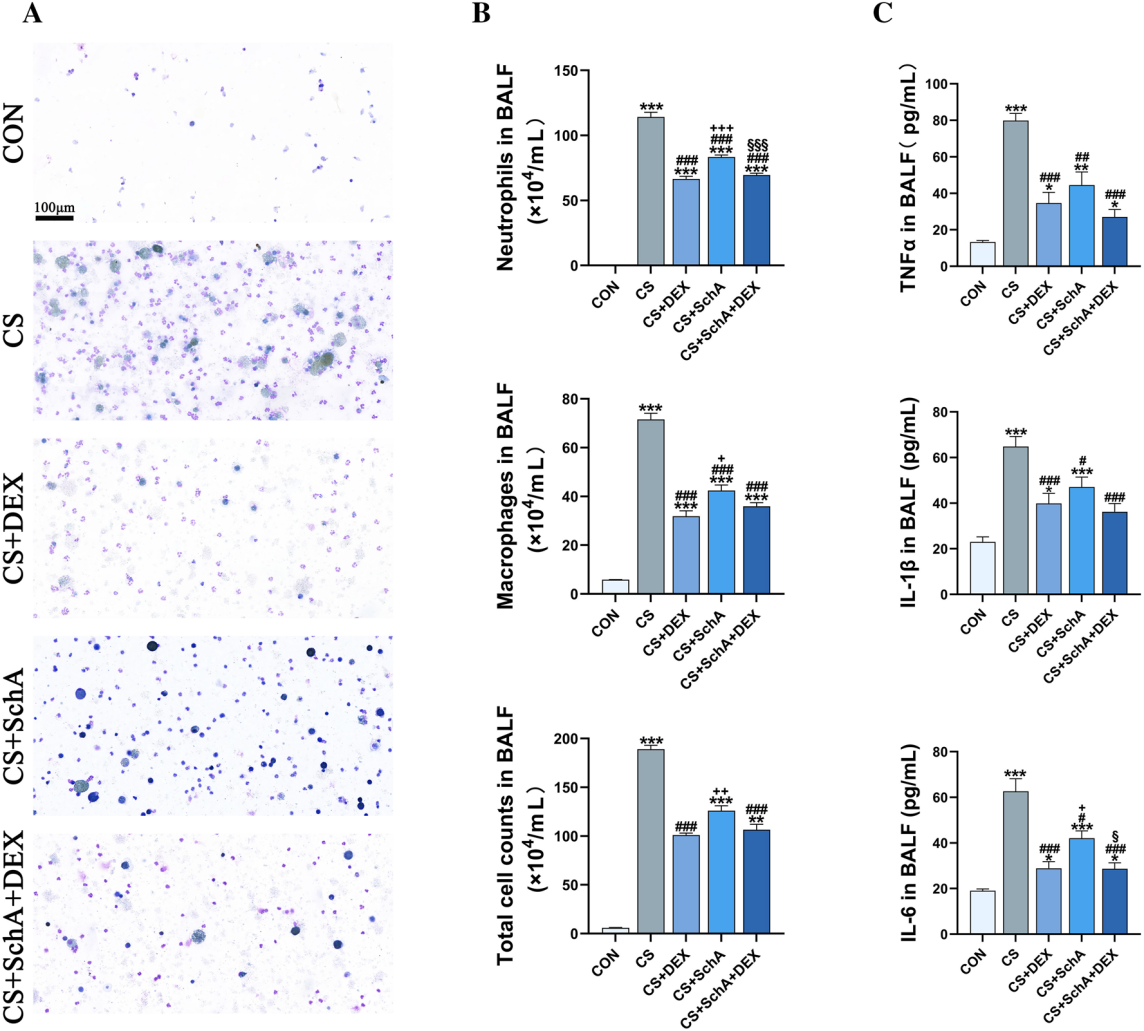
SchA Ameliorates Inflammatory Cells and Factors in the BALF of CS-induced COPD Model Mice. **A** Representative images of leukocytes in BALF from each group (scale bar = 100 μm). **B** The numbers of total cells, macrophages and neutrophils in BALF. **C** The levels of IL-6, IL-1β and TNF-α in BALF were measured. *n* = 6 mice per group. Data are shown as the mean ± SEM (**B** Neutrophils and Macrophages in BALF, **C**) and median ± IQR (**B** Total cell counts in BALF). To test for group differences, Games–Howell (**B** Neutrophils and Macrophages in BALF, C IL-6 and TNF-α), Tukey (**C** IL-1β) and Kruskal‒Wallis (**B** Total cell counts in BALF) tests were used. *p < 0.05, **p < 0.01, ***p < 0.001 versus control group, ^#^*p* < 0.05, ^##^*p* < 0.01, ^###^*p* < 0.001 versus CS group, ^+^*p* < 0.05, ^++^*p* < 0.01, ^++^*p* < 0.001 versus CS + DEX group, ^§^*p* < 0.05, ^§§^*p* < 0.01, ^§§§^*p* < 0.001 versus CS + SchA group

To study the regulatory effect of SchA on airway inflammation, we collected BALF, counted inflammatory cells, and measured the levels of the inflammatory factors TNF-α, IL-1β and IL-6. First, we analyzed the total number of inflammatory cells in the BALF of each group (Fig. 4).

We found that the total number of inflammatory cells in the BALF of the CS group was significantly higher than that of the control group, while Dex treatment and combination treatment significantly reduced the total number of inflammatory cells in the BALF of COPD model mice. Furthermore, CS mainly triggers the recruitment of many neutrophils and macrophages to engulf soot particles, which was reduced by DEX and SchA treatment individually. Moreover, it appeared that SchA + DEX treatment had marked efficacy in inhibiting the recruitment of inflammatory cells, similar to the DEX group.

Then, we analyzed the effect of SchA treatment on cytokine secretion in CS-exposed mice. Consistently, DEX or SchA treatment alone significantly reduced CS-induced TNF-α, IL-1β and IL-6 cytokine secretion. Although SchA treatment has a weaker effect on cytokine reduction than DEX, it still has a significant therapeutic effect when compared to cytokine release in the CS group. In addition, the efficacy of SchA and Dex combination therapy does seem to show a better result in descending the inflammatory factors. This may be due to the effect of SchA.

### SchA treatment reduces oxidative stress in CS-induced COPD model mice

**Fig. 5:**
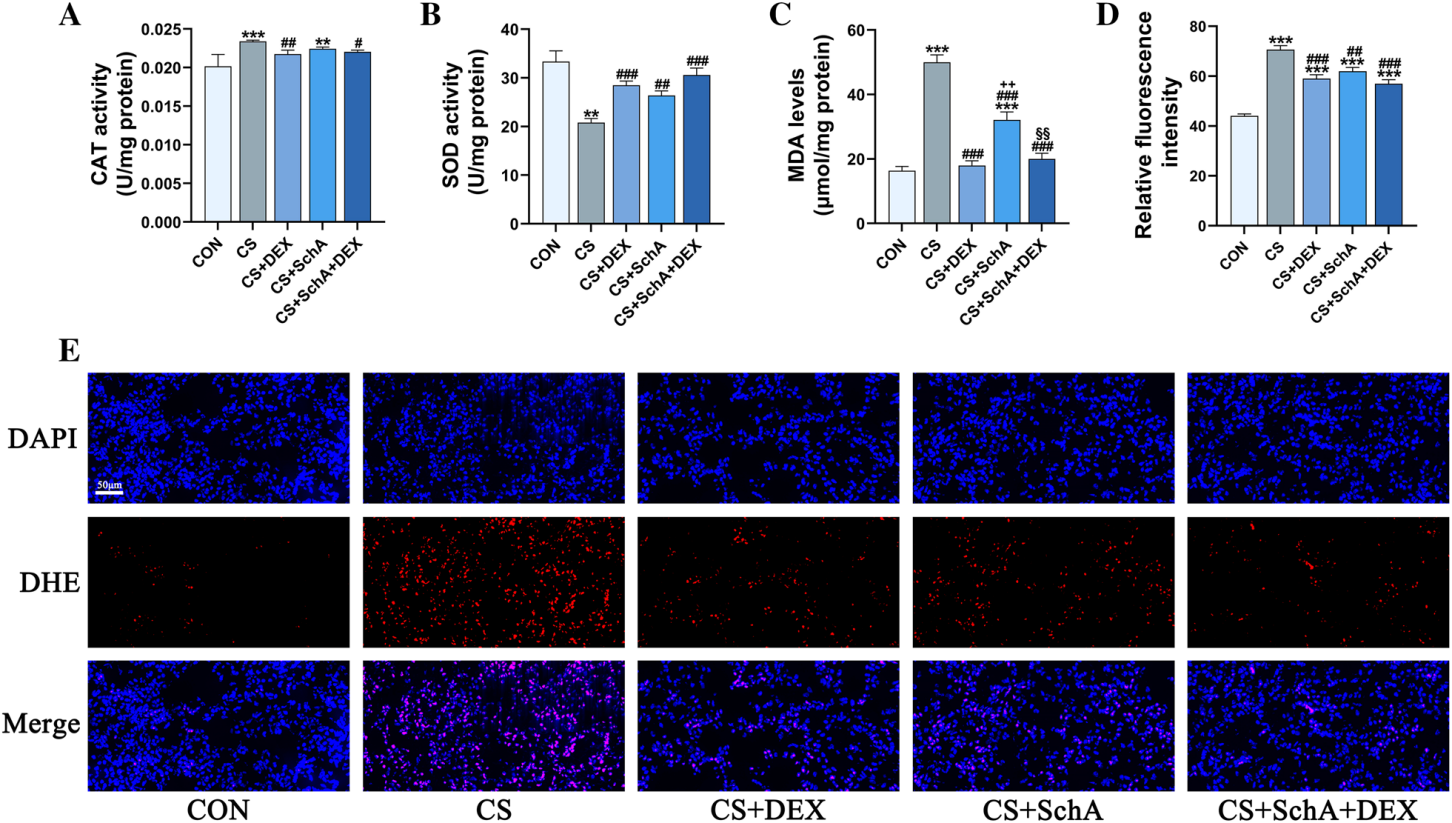
SchA Treatment Reduces Oxidative Stress in CS-induced COPD Model Mice. The activity of **A** CAT and **B** SOD and the levels of **C** MDA in the right lung tissue. **D** The relative DHE fluorescence intensity in lung tissues in the different groups. **E** Representative images of lung sections stained with DHE (rad) and DAPI (blue) (scale bar = 50 μm). *n* = 6 mice per group. Data are shown as the mean ± SEM (**B**–**D**) and median ± IQR (**A**). To test for group differences, Games–Howell (**B**–**D**) and Kruskal‒Wallis (**A**) tests were used. *p < 0.05, **p < 0.01, ***p < 0.001 versus control group, ^#^*p* < 0.05, ^##^*p* < 0.01, ^###^*p* < 0.001 versus CS group, ^+^*p* < 0.05, ^++^*p* < 0.01, ^++^*p* < 0.001 versus CS + DEX group, ^§^*p* < 0.05, ^§§^*p* < 0.01, ^§§§^*p* < 0.001 versus CS + SchA group

To further study the antioxidant activity of SchA in CS-induced inflammation, we measured the activity of superoxide dismutase (SOD) and catalase (CAT) and the levels of malonaldehyde (MDA) in lung tissue. For oxidative stress damage, we labeled DHE with fluorescence and performed quantitative and qualitative analyses to determine ROS levels. As shown in Fig. 5a–c, we found that the CAT activity and MDA content in the lung tissue of CS-induced COPD mice were significantly higher than those of the control group, while SOD activity was significantly reduced. SchA and Dex treatment can markedly reverse the CS-induced changes in antioxidant activity. In the analysis of the ROS level (Fig. 5d, e), we also observed a similar phenomenon. In CS-induced COPD model mice, the red fluorescence of DHE representing ROS was significantly increased, indicating higher ROS, and SchA and Dex treatment apparently downregulated it.

### SchA treatment upregulates the Nrf2 pathway in CS-induced COPD model mice

**Fig. 6:**
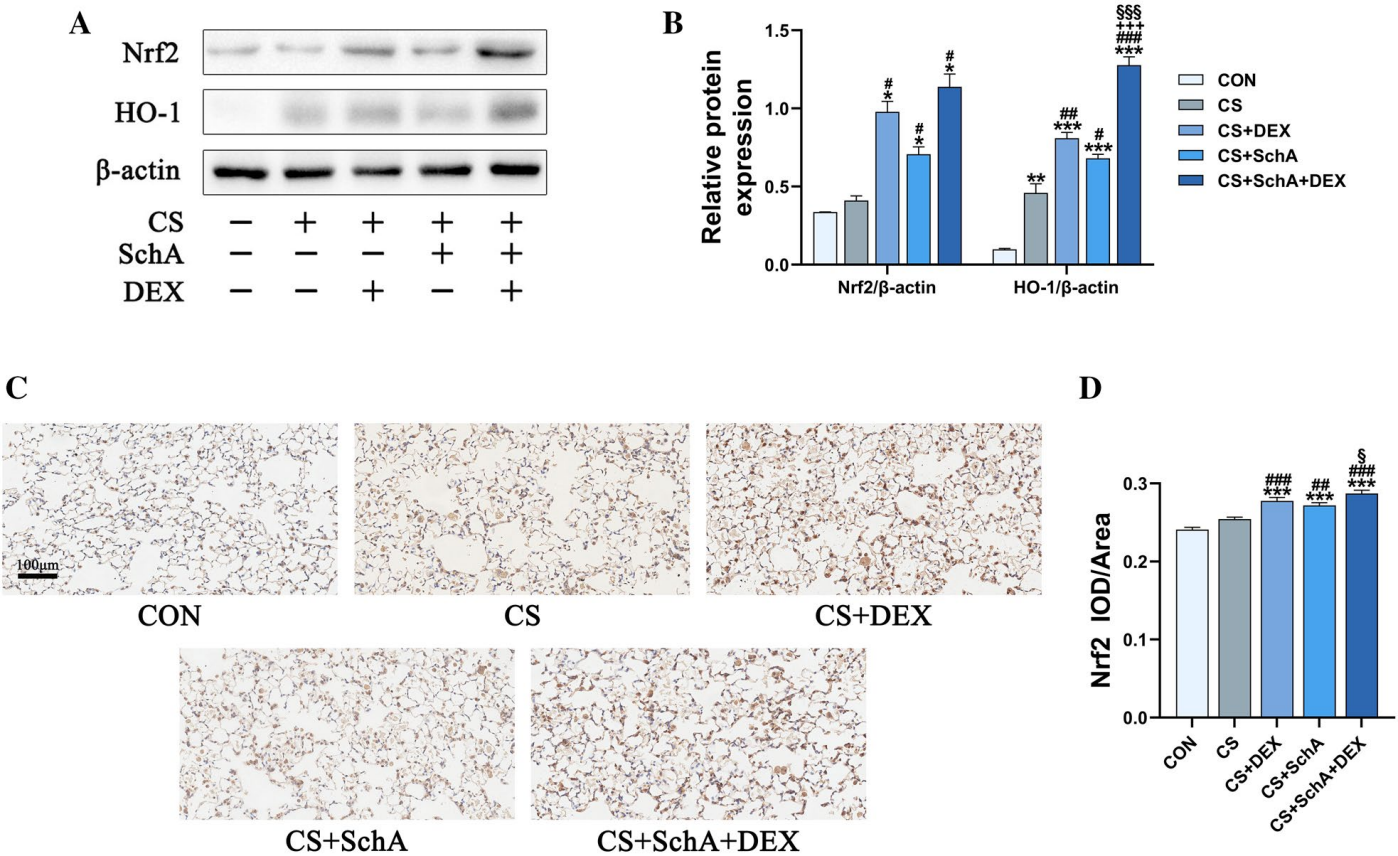
SchA Treatment Upregulates the Nrf2 Pathway in CS-induced COPD Model Mice. **A** The protein expression levels of Nrf2 and HO-1 were measured by Western blotting. β-Actin was used as the internal reference. **B** The band intensities of Nrf2 and HO-1 were semiquantified using ImageJ software. **C** Detection of Nrf2 levels in lung tissue by immunohistochemistry (scale bar = 100 μm). **D** Quantitative analysis statistics of Nrf2 in immunohistochemistry.* n* = 3 mice per group (**A**, **B**)*, n* = 6 mice per group (**C**, **D**). Data are shown as the mean ± SEM (**B**, **D**). To test for group differences, Games–Howell (**B** Nrf2/β-Actin) and Tukey (**B** HO-1/β-Actin, **D**). *p < 0.05, **p < 0.01, ***p < 0.001 versus control group, ^#^*p* < 0.05, ^##^*p* < 0.01, ^###^*p* < 0.001 versus CS group, ^+^*p* < 0.05, ^++^*p* < 0.01, ^++^*p* < 0.001 versus CS + DEX group, ^§^*p* < 0.05, ^§§^*p* < 0.01, ^§§§^*p* < 0.001 versus CS + SchA group

Next, we explored the potential mechanism by which SchA treatment inhibits inflammation in COPD model mice. The Nrf2 pathway has been widely reported to play a major role in COPD inflammation [20]. As shown in Fig. 6, we measured the expression levels of Nrf2 and HO-1 and found that SchA treatment significantly upregulated their expression levels as well as DEX treatment. Compared with the control group and CS group mice, the expression of Nrf2 protein was upregulated in SchA, Dex and combination treatment group mice (Fig. 6a, b). The protein expression of HO-1 was also higher than that in the control group and the CS group after treatment, especially in the SchA + DEX combination treatment groups (Fig. 6a, b). We also obtained similar results in immunohistochemistry of Nrf2 (Fig. 6c, d). SchA, Dex, and the combined treatment significantly increased the level of Nrf2 in lung tissues.

### SchA treatment inhibits NLRP3 pathway interference with cell pyroptosis in CS-induced COPD model mice

This study explored the effects of SchA on NLRP3 and its downstream molecules to determine the potential mechanism by which SchA treatment alleviates COPD symptoms. CS-induced lung injury leads to oxidative stress, and oxidative stress further increases NLRP3 inflammasome activation. Our results further confirm this phenomenon.

**Fig. 7:**
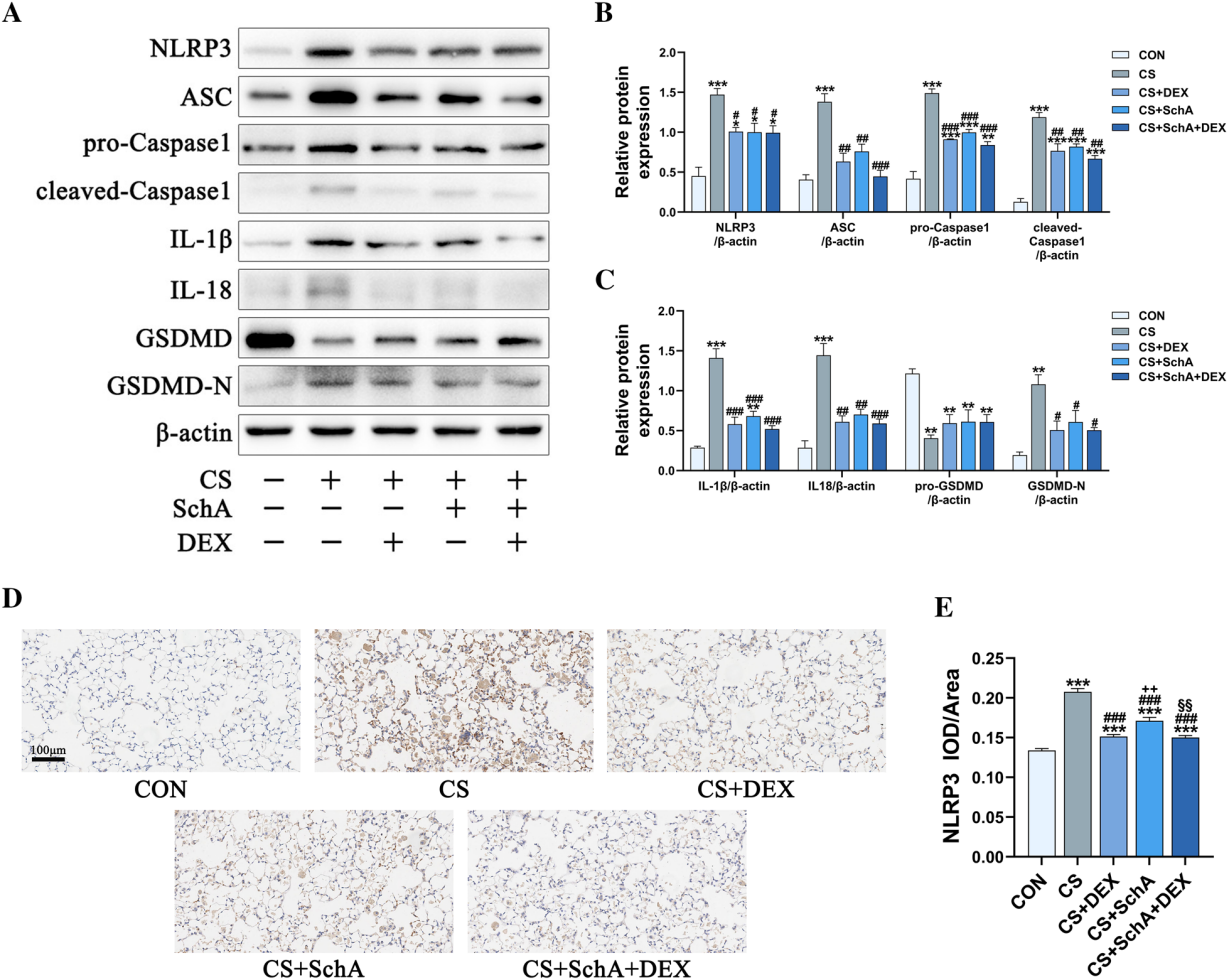
SchA Treatment Inhibits NLRP3 Pathways in CS-induced COPD Model Mice. **A** The protein expression levels of NLRP3, ASC, pro-Caspase1, cleaved-Caspase1 IL-1β, IL-18, GSDMD and GSDMD-N were measured by Western blotting. β-Actin was used as the internal reference. **B** The band intensities of NLRP3, ASC, pro-Caspase1, cleaved-Caspase1 IL-1β, IL-18, GSDMD and GSDMD-N were semiquantified using ImageJ software. **C** Detection of NLRP3 levels in lung tissue by immunohistochemistry. **D** Quantitative analysis statistics of NLRP3 in immunohistochemistry. *n* = 3 mice per group (**A**–**C**), *n* = 6 mice per group (**D**, **E**). Data are shown as the mean ± SEM (**B**, **C**, **E**). To test for group differences, Games–Howell (**E**) and Tukey (**B**, **C**) tests were used. *p < 0.05, **p < 0.01, ***p < 0.001 versus control group, ^#^*p* < 0.05, ^##^*p* < 0.01, ^###^*p* < 0.001 versus CS group, ^+^*p* < 0.05, ^++^*p* < 0.01, ^++^*p* < 0.001 versus CS + DEX group, ^§^*p* < 0.05, ^§§^*p* < 0.01, ^§§§^*p* < 0.001 versus CS + SchA group

Compared with the control group, the levels of NLRP3, ASC, and pro-Caspase1 in the CS group were significantly higher, while Dex or SchA treatment inhibited the expression of proteins related to this inflammatory protein complex (Fig. 7a, b), and the immunohistochemistry results were also consistent (Fig. 7d, e). CS exposure significantly increased the expression of the downstream inflammatory molecules IL-1β and IL-18, but Dex and SchA treatment inhibited this effect (Fig. 7a, c). Moreover, GSDMD is a protein naturally existing in cells. Under oxidative stress conditions, GSDMD converts into GSDMD-N and forms holes in the cell membrane, leading to pyroptosis. From our WB experimental results, Dex and SchA treatment can inhibit this process.

## Discussion

To our knowledge, this study is the first animal experimental study in the treatment of COPD induced by CS with SchA. Our results show that SchA treatment can improve lung function and ameliorate COPD symptoms by significantly reducing the excessive secretion of proinflammatory cytokines, inflammatory cell infiltration and emphysema in CS-induced COPD mice. In addition, we explored the mechanism by which SchA exerts its anti-inflammatory effects on COPD model mice and found that SchA may modulate the Nrf2 signaling pathway and NLRP3 inflammasome activation, which may be significant in the treatment of COPD.

Similar to Dex treatment, SchA treatment significantly reversed the decline in lung function. CS exposure can cause significant weight loss, which we used as a sign of successful modeling. Other scholars found that Dex treatment can further reduce the weight of mice after CS exposure [14]. This is mainly because the body weight of mice after Dex treatment shows a downward trend, which may be related to the decrease in protein synthesis, increase in protein decomposition, and slow growth of muscles and other tissues after the application of large concentrations of glucocorticoids. However, our results did not find any significant difference in weight between the SchA and Dex treatment groups, which we believe may be related to the different administration methods of Dex we used. We did not use the early high-dose administration method similar to other studies, so we speculate that this is the reason body weight did not further decrease with Dex treatment.

Pulmonary function is more sensitive than lung morphological changes and is considered the most important auxiliary examination in the diagnosis of COPD, and small airway lesions can be observed in COPD patients [21]. Our data showed that the mice exposed to CS showed significant airflow restriction and malignant lung expansion, decreased FEV50/FVC values, and increased FVC, RI and chord compliance values. Impressively, after treatment with SchA, we observed that the impaired lung function of CS-induced COPD model mice was significantly ameliorated. However, it should also be noted that the therapeutic effect of SchA is not better than that of Dex. As a classical drug for treating COPD, Dex significantly improved the lung function of CS-induced COPD mice.

Compared with the control group, SchA treatment significantly reduced the infiltration of inflammatory cells, the secretion of proinflammatory cytokines and lung pathological damage, which is similar to the treatment effect of Dex. Abnormally activated monocytes/macrophages and neutrophils are often considered the core of the inflammatory response in COPD. They produce proinflammatory mediators, such as IL-6, IL-1β and TNF-α. Moreover, inflammatory cells in the lungs of patients with COPD accumulate in the lung and show a persistent inflammatory state, which leads to further destruction of the alveolar structure and decline in lung function [22]. Some scholars have confirmed that cytokines, mainly IL-6 secreted by macrophages, have strong neutrophil chemotaxis and have been confirmed to be related to the severity of COPD, the rate of decline in lung function, and the progression of emphysema [23, 24]. In addition, IL-1β is also an important cytokine in the inflammatory reaction [25, 26]. Moreover, IL-6, as an important marker of inflammation, is also associated with the systemic manifestations of COPD [27]. Tumor necrosis factor (TNF-α) is a multieffect cytokine with several functions, such as growth promotion, growth inhibition, angiogenesis, cytotoxicity, inflammation and immune regulation, which are involved in a variety of inflammatory conditions. It plays an important role in many pulmonary inflammatory diseases. Many studies support the hypothesis that TNF-α plays an important pathobiological role in lung diseases, including severe refractory asthma and COPD. In our study, we also confirmed that SchA treatment can effectively reduce the levels of IL-6, IL-1β and TNF-α. This reduction in cytokines is also one of the reasons for the improvement in COPD symptoms.

SOD is an important enzyme that restrains oxidative stress [28]. It is widely distributed in the biological world, from animals to plants and even from humans to single-celled organisms. SOD is regarded as a scavenger of free radicals in the human body [29]. In our study, we found that administration of Dex and SchA can effectively increase the level of SOD, which is of great significance for the treatment of COPD. CAT exists in red blood cells and peroxides in some tissues. Its main role is to catalyze the decomposition of H_2_O_2_ into H_2_O and O_2_ so that H_2_O_2_ will not react with O_2_ under the action of iron chelates to generate very harmful OH. Almost all biological organisms have CAT. However, in our study, CAT was increased in the CS group, which is considered the result of a self-protection effect. More importantly, after treatment, the levels of CAT and SOD increased. The enzymatic activity of SOD and CAT provides an antioxidant defense mechanism for the body. Free radicals act on lipid peroxidation, and the end product of oxidation is MDA, which causes cross-linking and polymerization of biological macromolecules such as proteins and nucleic acids and is cytotoxic. MDA is one of the most important products of membrane lipid peroxidation [30, 31], and its production can also damage the cell membrane. We found that SchA reduced the level of MDA. Moreover, DHE staining intuitively shows the severity of oxidative stress [32]. In our qualitative and quantitative studies, we found that SchA plays an important role in reducing oxidative stress.

Many scholars have reported that the Nrf2/HO-1 pathway protects cells from oxidative stress [33–35]. This study confirmed that SchA can modulate the Nrf2/HO-1 pathway. The role of the Nrf2 signaling pathway in COPD airway inflammation has been widely confirmed as a protective protein after injury. An increasing number of studies have reported that the activation of Nrf2 has a positive effect on the treatment of COPD inflammation [36]. Some scholars believe that CS exposure leads to a reduction in Nrf2 [14].

Conversely, other scholars believe that the lung tissue product Nrf2 responds to CS exposure [37] since Nrf2 is a transcription factor that regulates the expression of genes involved in cellular defense mechanisms, including antioxidant and anti-inflammatory pathways. Similarly, in our study, we found that CS exposure caused lung tissue damage in mice, which led to an increase in Nrf2 levels. Furthermore, our results illustrated that SchA and Dex treatment markedly stimulated the production of Nrf2, which indicated that the increased Nrf2 level can effectively reverse oxidative stress. The activation of Nrf2 not only alleviates the inflammatory response of COPD by inducing HO-1 but also prevents elevated levels of proinflammatory mediators, including IL-6 [38]. More importantly, SchA treatment also upregulated protective factors downstream of Nrf2, such as HO-1, which may explain the anti-inflammatory effect of SchA on CS-induced COPD. Our research has some limitations, such as not investigating whether the regulation of SchA in Nrf2 signaling plays an anti-inflammatory role by reducing oxidative stress, although Nrf2 is indeed related to oxidative stress in other scholars' reports. However, our study does indicate that SchA has the same anti-inflammatory effect as Dex in the treatment of COPD model mice, and we have not observed any obvious side effects of SchA treatment. The high safety of SchA may make it a potential candidate drug for COPD, especially when Dex treatment is unavailable or not appropriate in certain clinical circumstances.

In cell biology, cell death has always been a research hotspot. Cell pyroptosis, also known as inflammatory necrosis of cells [39–41], is a new proinflammatory and programmed cell death mode that was recently discovered [7]. The activation of cell pyroptosis involves different caspases, which are mainly divided into the classical cell pyroptosis pathway depending on Caspase-1 and the nonclassical cell pyroptosis pathway depending on Caspase-4/5/11 [42]. Caspase-1 is an important part of the NLRP3 inflammasome.

When cells are stimulated by exogenous or endogenous stimuli, such stimulation will promote the expression of NLRP3 in cells [43]. In short, the intracellular NOD-like receptor (NLR) recognizes these signals, activates the assembly of the inflammasome complex, and recruits pro-Caspase-1 to bind to the inflammasome complex through the adaptor protein ASC [44]. The local concentration of the recruited pro-Caspase-1 increases, and autogenous splicing occurs in Caspase-1 and enzymatically activates pro-IL-1β and pro-IL-18. Moreover, the activation of Caspase-1 mediates the subsequent activation of Gasdermin D (GSDMD) protein, which is the key mediator of cell death [42]. In our research, we found that the expression of NLRP3 in the lung tissue of the CS group was significantly higher than that in the control group, and the expression of ASC and pro-Caspase-1 increased synchronously. We speculated that the CS group had produced obvious inflammasome complexes at this time. This finding is similar to the results of other scholars studying long-term CS exposure in mice [45]. In turn, SchA significantly attenuated the expression of the NLRP3 inflammasome and downstream molecules, while DEX and SchA + DEX also showed better efficacy. More importantly, the Nrf2 pathway has been shown to regulate the activation of the NLRP3 inflammasome, thereby modulating the inflammatory response and preventing pyroptosis.

Furthermore, we observed the expression of GSDMD, which has two conserved domains [46]: an N-terminal effect domain and a C-terminal inhibition domain. The N-terminus is the main functional domain. The N-terminus can participate in the occurrence of pyroptosis, while the C-terminus functions in autoinhibition. Under stimulation, for example, oxidative stress, activated Caspase-1 cleaves the GSDMD protein to induce cell pyroptosis [47, 48]. GSDMD-N can target the phospholipid protein on the cell membrane, polymerize and form holes on the cytoplasmic membrane, which leads to cell pyroptosis [49]. In addition, activated IL-1β and IL-18 are released out of the cells through the pores, leading to the occurrence of inflammatory reactions [50]. In our study, we also found that compared with other experimental groups, the expression of GSDMD was the highest in the lung tissue of healthy mice. CS exposure causes GSDMD to be activated into GSDMD-N. Moreover, SchA treatment inhibited this process and reduced GSDMD. Similarly, Dex treatment also inhibits this process. We believe that SchA can be used as a new alternative drug in the treatment of COPD.

Interestingly, in our results, the combination of DEX and SchA did not show objective advantages in the results. We believe that this may be due to the mechanism of action of SchA being similar to that of DEX and the inhibitory effect of inflammation and pyroptosis caused by the same pathway. Therefore, the combination of the two drugs has no synergistic effect; this may be an 'occupying' effect, but of course, further research is needed to confirm it.

In summary, this study discussed the efficacy of SchA in the treatment of COPD caused by CS and compared it with Dex and further investigated its underlying therapeutic mechanism. It has been proven that SchA plays a role in the treatment of COPD in lung function, as observed in the BALF, pathology and others of COPD model mice. However, we found that the therapeutic effect of SchA is clearly weaker than that of Dex in some respects. Notably, if Dex cannot be used under certain circumstances, that is, if it cannot be used due to the contraindication of Dex or other reasons, SchA can be used as an alternative drug, which is of great clinical significance.

## Conclusion

Our research shows that SchA treatment significantly inhibits the inflammatory response in CS-induced COPD model mice, mainly by affecting the Nrf2/NLPR3 pathway.

## Data Availability

The data sets are not publicly available due to restrictions used under the license for the current study. However, they are available on reasonable request from the corresponding author.
